# Anxiety, depression, and coping styles among patients with chronic pancreatitis in East China

**DOI:** 10.1186/s12888-023-04691-2

**Published:** 2023-03-29

**Authors:** Cui Chen, You Zhou, Dan Wang, Ge Li, Kun Yin, Hong Tao, Chun-Yan Wang, Zhao-Shen Li, Cun Wei, Liang-Hao Hu

**Affiliations:** 1 Department of Gastroenterology, First Affiliated Hospital of Naval Medical University, No. 168 Changhai Road, Yangpu District, Shanghai, China; 2 Department of Nursing, Affiliated Hospital of Yangzhou University, Yangzhou University, Yangzhou, China; 3 Department of Naval Psychology, Faculty of Psychology, Naval Medical University, No. 800 Xiangyin Road, Yangpu District, Shanghai, China

**Keywords:** Mental health, Anxiety, Depression, Chronic pancreatitis, Risk factors

## Abstract

**Background:**

Anxiety and depression are common psychological comorbidities in patients with chronic pancreatitis (CP). There is still a lack of epidemiological studies on anxiety and depression in Chinese CP patients. This study aimed to identify the incidence and related factor of anxiety and depression among East Chinese CP patients and explore the relationship between anxiety, depression, and coping styles.

**Methods:**

This prospective observational study was conducted from June 1, 2019 to March 31, 2021 in Shanghai, China. Patient diagnosed with CP were interviewed using the sociodemographic and clinical characteristics questionnaire, Self-rating Anxiety Scale (SAS), Self-rating Depression Scale (SDS), and Coping Style Questionnaire (CSQ). Multivariate logistic regression analysis was conducted to identify the related factors of anxiety and depression. Correlation test was preformed to analyze the correlation between anxiety, depression, and coping styles.

**Results:**

The incidence of anxiety and depression in East Chinese CP patients was 22.64% and 38.61%, respectively. Patients’ previous health status, level of disease coping, frequency of abdominal pain episodes, and pain severity were significantly associated with anxiety and depression. Mature coping styles (Problem solving, Seeking for help) had a positive impact on anxiety and depression, while immature coping styles (Self-blame, Fantasy, Repression, Rationalization) had negative effects on anxiety and depression.

**Conclusion:**

Anxiety and depression were common in patients with CP in China. The factors identified in this study may provide references for the management of anxiety and depression in CP patients.

## Introduction

Chronic pancreatitis (CP) is a progressive inflammatory disease characterized by irreversible pancreatic fibrosis [1]. Current studies suggested that the development of CP is associated with the interaction between environmental (tobacco, alcohol, etc.) and genetic factors [2–4]. Globally, the incidence of CP ranges from 4.4 to 14 per 100,000 people, the prevalence ranges from 36.9 to 52.4 per 100,000 people, and the mortality rate is about 0.09 per 100,000 persons [5, 6]. The incidence of CP is significantly different in different regions. In the United States [7], the UK [8], China [9], Denmark [10], and Japan [11], the annual incidence of CP was about 5 to 8, 5.9 to 12.8, 3.1 to 13.5, 12.6, and 14.0 per 100,000 persons, respectively. Although there is still a lack of large-scale epidemiological studies on the incidence and prevalence of CP currently, the available data have shown that, with the increases of alcohol consumption and the application of more sensitive imaging technologies in recent years, the incidence of CP is increasing [10, 12].

Pain is the most common symptom of CP and the main reason for patients to seek medical attention, and more than 80% patients with CP will experience varying degrees of upper abdominal pain during the course of the disease [13]. Due to the atrophy of pancreatic acinar cells and pancreatic fibrosis, patients with CP also often experience obvious symptoms of pancreatic insufficiency such as elevated blood sugar, weight loss, sarcopenia, and complications including pancreatic duct stones, pancreatic duct stenosis, pancreatic pseudocysts and pancreatic cancer, which affect the quality of life seriously [7]. Pain, decline in quality of life, and concerns about the prognosis of the disease can bring enormous psychological stress for CP patients and undermine their confidence in coping with disease, ultimately leading to the occurrence of psychiatric comorbidities [14].

As the most common psychological complications in hospitalized patients, anxiety and depression among CP population have received increasing attention from researchers in recent years. A recent multicenter study conducted in the United States and Denmark showed that the incidence of anxiety and depression among CP patients was 46.8% and 38.6%, respectively, and there were significant differences in pain patterns, pain severity, and quality of life between CP patients with and without psychological comorbidities [14]. Furthermore, in India, a study reported that 34.85% of CP patients had anxiety symptoms and 29.23% had depression symptoms [15]. However, at present, there are no studies related to the incidence and influencing factors of anxiety and depression symptoms in Chinese CP patients. Therefore, the aims of this study were (1) to investigate the incidence and level of anxiety and depression symptoms among East Chinese patients with CP; (2) to analyze the factors associated with anxiety and depression, and to explore the relationship between anxiety, depression, and coping styles.

## Materials and methods

### Research Methods and Recruitment Criteria

This was a prospective observational study conducted from June 1, 2020 to June 31, 2021, which recruited patients diagnosed with CP according to the 2018 Chinese guidelines for the diagnosis and treatment of chronic pancreatitis [5] and hospitalized in the Department of Gastroenterology at First Affiliated Hospital of Naval Medical University, Shanghai, the largest CP treatment center in China.

The study population included all adult patients who met the diagnostic criteria. The exclusion criteria were as follows: patients (1) were illiterate; (2) had communication impairment and were unable to complete the questionnaires; (3) had a history of psychiatric disorders and recent use of psychotropic and sedative drugs; (4) had malignancy; and (5) refused to participate in this study. For those with multiple admission records, we selected only the records from the first admission for analysis.

The protocol of this study was approved by the ethical committees of the Naval Medical University (CHEC2020-026), and the study was conducted in accordance with the Declaration of Helsinki. All participants signed an informed consent.

### Measurements

#### Sociodemographic and clinical characteristics Questionnaire

The researchers designed questionnaires on the sociodemographic and clinical characteristics of the participants through a literature review. The sociodemographic questionnaire included the following seven items: age, gender (male or female), education level (primary school, junior high school–senior high school, university or above), marital status (single or divorced or widowed, married), perceived support from family and friends (low or moderate, high), previous health status (poor, moderate and good) and subjective disease coping level (poor, moderate and good).

The clinical characteristics questionnaire of CP included the following four items: pain as a first symptoms (yes or no), frequency of abdominal pain during the last 12 months (< 3 times, ≥ 3 times, constant pain), visual analogue scale of pain (VAS-P) (0–2 points, 3–6 points, 7–10 points), and times of hospitalization of acute attack of CP (< 3 times, ≥ 3 times).

#### The self-rating anxiety scale

Anxiety symptoms of patients were assessed using the Zung Self-Rating Anxiety Scale (SAS), which includes 20 items with possible answers on a 4-point Likert scale (1= “none or little of the time”; 2= “some of the time”; 3= “good part of the time”; 4= “most of the time”) to reflect the intensity of anxiety over the past week or two [16, 17]. The scale includes 15 items associated with increased anxiety and five items associated with decreased anxiety, with raw scores ranging from 20 to 80. The higher the score, the greater the intensity of the patient’s anxiety. The standardized score (1.25* raw score) ranging from 50 to 59, 60 to 69, and 70 or more are considered mild, moderate, and severe anxiety, respectively [18]. The Chinese version of the SAS has been validated in the Chinese population with a Cronbach’s alpha coefficient of 0.73.

#### The self-rating Depression Scale

Depression symptoms were assessed using the Zung Self-rating Depression Scale (SDS) [19]. Similar to the SAS, the SDS is also a patient self-assessment tool to reflect the degree of depression and contains 20 items, which has the same scoring method as the SAS. The scale includes 10 items associated with increased and decreased depression, respectively. The higher the score, the higher the degree of depression of the patient. The standardized score (1.25* raw score) of 53 to 62, 63 to 72, and 72 or more are considered mild, moderate, and severe depression, respectively [20]. The Cronbach’s alpha coefficient of Chinese version of the SDS was 0.79.

#### Coping Style Questionnaire

The coping style of patients with CP was assessed using the Chinese Coping Style Questionnaire (CSQ) compiled by Xiao et al. in 1996 [21]. The CSQ was consisted of six dimensions: problem solving, self-blame, seeking for help, fantasy, repression, and rationalization, with a total of 62 items. Each item has two answers, yes and no, rated with 1 and 0 points, respectively. The score of each dimension is the ratio of the total score of items to the number of items in that dimension. The higher the score for the dimension, the more likely the participant is to adopt this coping style. Of the six dimensions, problem solving and seeking for help are considered mature coping styles, self-blame, fantasy, repression, and rationalization are considered immature coping style [22]. The reliability and validity of the CSQ has been validated, and the instrument is widely used in China.

### Statistical analysis

All sociodemographic and disease-related data, as well as the incidence of anxiety and depression among Chinese CP patients, were analyzed descriptively. Categorical data were expressed as frequencies and percentages, and continuous data were expressed as means and standard deviations (SD). The Kolmogorov-Smirnov test was used for normality of continuous data, and the Pearson χ2 test, Student’s t-test, and nonparametric Mann-Whitney test were used to compare the between-group differences in categorical data, and continuous data with normal and skewed distributions, respectively. Multivariate logistic regression analysis was performed to identify independent related factors of anxiety and depression among Chinese CP patients. Pearson correlation test was conducted to analyze the correlation between the score of anxiety, depression, and coping styles. Statistical analyses were performed using the SPSS software version 26.0. All statistics were tested using a two-sided test, and a p-value less than 0.05 was considered statistically significant.

## Results

### Characteristics of participants

Table 1 presented the characteristics of the participants. A total of 720 patients were included in this study. In terms of sociodemographic data, the mean age of the patients was 42.74 ± 22.56 years, of whom 30.56% were female, 77.92% were married, and 43.33% received university or higher education. 58.33% of the participants claimed they previously had good health status, and almost all subjects (93.75%) reported that they received a high level of support from family and friends. 60.42% believed they had a good response to the disease. Data on the clinical characteristics of CP showed that 82.22% of participants had abdominal pain as their first symptom, and more than half (56.81%) of patients were hospitalized three or more times for acute attack of CP. As for the frequency of abdominal pain, 32.92% reported they had more frequent (≥ 3 times) or persistent abdominal pain in the last 12 months. As for the severity of pain, 63.47% of patients had moderate to severe pain (VAS-P > 3 points). In addition, the score of CSQ in problem solving, self-blame, seeking for help, fantasy, repression, and rationalization was 0.79 ± 0.21, 0.33 ± 0.29, 0.58 ± 0.22, 0.48 ± 0.23, 0.47 ± 0.23, 0.48 ± 0.20, respectively.

**Table 1:** Characteristics of participants (n = 720)

Characteristics	n (%)
Age, y (mean [SD])	42.74 (22.56)
Female sex, n (%)	220 (30.56)
Educational level, n (%)	
Primary school	70 (9.72)
Junior high school - Senior High School	338 (46.94)
University or above	312 (43.33)
Marital status, n (%)	
Single/Divorced/Widowed	159 (22.08)
Married	561 (77.92)
Perceived support from family and friends, n (%)	
Low/Moderate	45 (6.25)
High	675 (93.75)
Previous health status, n (%)	
Poor	25 (3.47)
Moderate	275 (38.19)
Good	420 (58.33)
Subjective disease coping level, n (%)	
Poor	18 (2.50)
Moderate	267 (37.08)
Good	435 (60.42)
Times of hospitalization of acute attack of CP, n (%)	
< 3 times	311 (43.19)
≥ 3 times	409 (56.81)
Pain as first symptoms, n (%)	592 (82.22)
Frequency of abdominal pain during the last 12 months, n (%)	
< 3 times	483 (67.08)
≥ 3 times	219 (30.42)
Constant pain	18 (2.50)
VAS-P, n (%)	
0–2 points	263 (36.53)
3–6 points	257 (35.69)
7–10 points	200 (27.78)
Anxiety Symptoms (SAS)	
SAS score, (mean [SD])	43.11 (9.39)
No anxiety, n (%)	557 (77.36)
Mild anxiety, n (%)	126 (17.50)
Moderate anxiety, n (%)	30 (4.17)
Severe anxiety, n (%)	7 (0.97)
Depression Symptoms (SDS)	
SDS score, (mean [SD])	49.26 (11.02)
No depression, n (%)	442 (61.39)
Mild depression, n (%)	199 (27.64)
Moderate depression, n (%)	72 (10.00)
Severe depression, n (%)	7 (0.97)
Coping style (CSQ), (mean [SD])	
Problem solving	0.79 (0.21)
Self-blame	0.33 (0.29)
Seeking for help	0.58 (0.22)
Fantasy	0.48 (0.23)
Repression	0.47 (0.23)
Rationalization	0.48 (0.20)

Abbreviations: CP, chronic pancreatitis; CSQ, Coping Style Questionnaire; SAS, Self-rating Depression Scale; SD, standard deviations; SDS, Self-rating Depression Scale; VAS-P, visual analogue scale of pain

### Incidence of anxiety and depression

**Fig. 1:**
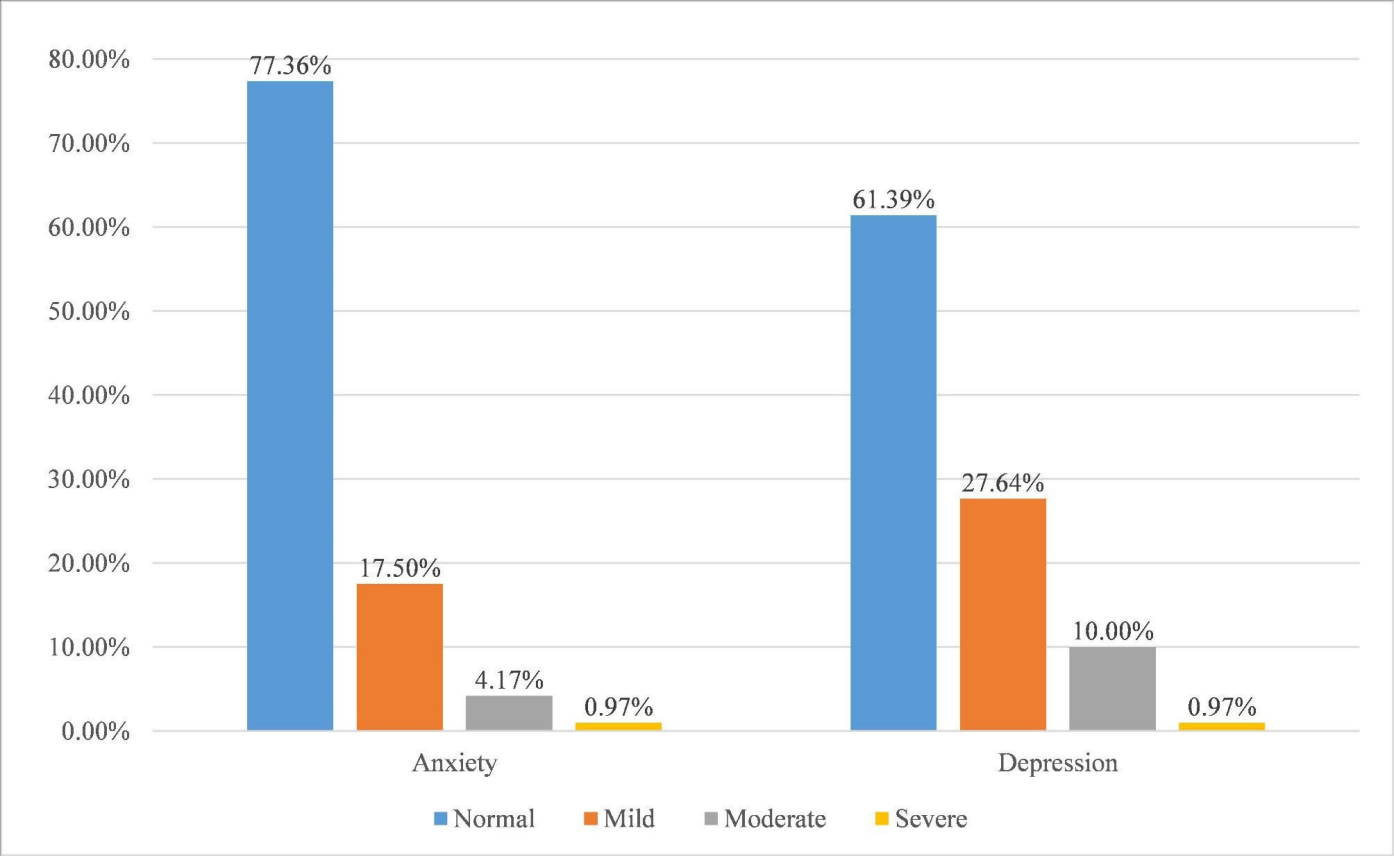
The percentage of anxiety and depression symptoms severity

Figure 1 showed the percentages of anxiety and depression symptom severity. The mean standard score of SAS in our cohort was 43.11 ± 9.39. The incidence of anxiety among Chinese CP patients was 22.64% (163/720), with mild, moderate and severe anxiety accounting for 17.50%, 4.17% and 0.97% of the total participants, respectively. The incidence of depression among Chinese CP patients was 38.61% (278/720), and the mean standard score of SDS was 49.26 ± 11.02. 27.64%, 10.00% and 0.97% of the total participants had mild, moderate and severe depression symptom, respectively (Table 1).

### Related factors associated with anxiety and depression

Table 2 presented the results of univariate analyses of anxiety, depression, and sociodemographic and clinical characteristics of the participants. Results showed that there were significant differences in age (P < 0.001), gender (P = 0.006), marital status (P < 0.001), previous health status (P < 0.001), level of subjective disease coping (P < 0.001), times of hospitalization (P = 0.002), first symptom (P = 0.011), frequency of abdominal pain (P < 0.001), and the severity of pain (P < 0.001) between patients with and without anxiety. Multivariable logistic regression analysis indicated that patients with moderate (P = 0.001) or poor (P = 0.003) previous health status were more likely to report anxiety than those with good previous health status, and compared to those with better subjective disease coping level, patients reported poor (P = 0.034) and moderate (P = 0.010) levels of subjective disease coping were more likely to report anxiety symptom. As for pain, constant pain (P = 0.002), and severe pain (VAS-P: 7–10 points) (P = 0.021) were considered to be significantly associated with the onset of anxiety. In addition, female (P = 0.039) was an independent risk factor for anxiety in patients with CP **(**Table 3**)**.

**Table 2:** Univariate analysis of anxiety, depression, and sociodemographic and clinical characteristics

		Anxiety				Depression			
Variables		No (n = 557)	Yes (n = 163)	*P* value		No (n = 442)	Yes (n = 278)	*P* value	
Age, y (mean [SD])		43.99 (24.30)	38.52 (14.51)	**< 0.001**		43.22 (14.47)	41.98 (31.32)	**0.012**	
Female sex, n (%)		156 (28.01)	64 (39.26)	**0.006**		126 (28.51)	94 (33.81)	0.132	
Educational level, n (%)									
Primary school		55 (9.87)	15 (9.20)	0.900		39 (8.82)	31 (11.15)	**0.025**	
Junior high school - Senior High School		259 (46.50)	79 (48.47)			194 (43.89)	144 (51.80)		
University or above		243 (43.63)	69 (42.33)			209 (47.29)	103 (37.05)		
Marital status, n (%)									
Single/Divorced/Widowed		105 (18.85)	54 (33.13)	**< 0.001**		93 (21.04)	66 (23.74)	0.395	
Married		452 (81.15)	109 (66.87)			349 (78.96)	212 (76.26)		
Perceived support from family and friends, n (%)									
Low/Moderate		30 (5.39)	15 (9.20)	0.077		18 (4.07)	27 (9.71)	**0.002**	
High		527 (94.61)	148 (90.80)			424 (95.93)	251 (90.29)		
Previous health status, n (%)									
Poor		11 (1.97)	14 (8.59)	**< 0.001**		6 (1.36)	19 (6.83)	**< 0.001**	
Moderate		193 (34.65)	82 (50.31)			155 (35.07)	120 (43.17)		
Good		353 (63.38)	67 (41.10)			281 (63.57)	139 (50.00)		
Subjective disease coping level, n (%)									
Poor		8 (1.44)	10 (6.13)	**< 0.001**		5 (1.13)	13 (4.68)	**< 0.001**	
Moderate		187 (33.57)	80 (49.08)			142 (32.13)	125 (44.96)		
Good		362 (64.99)	73 (44.79)			295 (66.74)	140 (50.36)		
Times of hospitalization of acute attack of CP, n (%)									
< 3 times		258 (46.32)	53 (32.52)	**0.002**		212 (47.96)	99 (35.61)	**0.001**	
≥ 3 times		299 (53.68)	110 (67.48)			230 (52.04)	179 (64.39)		
Pain as first symptoms, n (%)		447 (80.25)	145 (88.96)	**0.011**		348 (78.73)	244 (87.77)	**0.002**	
Frequency of abdominal pain during the last 12 months, n (%)									
< 3 times		394 (70.74)	89 (54.60)	**< 0.001**		324 (73.30)	159 (57.19)	**< 0.001**	
≥ 3 times		157 (28.19)	62 (38.04)			114 (25.79)	105 (37.77)		
Constant pain		6 (1.08)	12 (7.36)			4 (0.90)	14 (5.04)		
VAS-P, n (%)									
0–2 points		225 (40.39)	38 (23.31)	**< 0.001**		190 (42.99)	73 (26.26)	**< 0.001**	
3–6 points		191 (34.29)	66 (40.49)			145 (32.81)	112 (40.29)		
7–10 points		141 (25.31)	59 (36.20)			107 (24.21)	93 (33.45)		

Abbreviations: CP, chronic pancreatitis; SD, standard deviations; VAS-P, visual analogue scale of pain

**Table 3:** Multivariable logistic regression analysis of anxiety, depression, and sociodemographic and clinical characteristics

						95% CI	
Variables	*β*	SE	Wald	*P*	OR	Lower	Upper
**Anxiety**							
Age, y (mean [SD])	-0.013	0.009	2.422	0.120	0.987	0.970	1.003
Female sex, n (%)	0.425	0.205	4.280	**0.039**	1.529	1.023	2.287
Marital status, n (%)							
Married	Reference	Reference	Reference	Reference	Reference	Reference	Reference
Single/Divorced/Widowed	0.507	0.267	3.616	0.057	1.661	0.985	2.801
Previous health status, n (%)							
Good	Reference	Reference	Reference	Reference	Reference	Reference	Reference
Moderate	0.714	0.206	11.979	**0.001**	2.043	1.363	3.062
Poor	1.465	0.485	9.117	**0.003**	4.329	1.672	11.206
Subjective disease coping level, n (%)							
Good	Reference	Reference	Reference	Reference	Reference	Reference	Reference
Moderate	0.528	0.204	6.701	**0.010**	1.695	1.137	2.528
Poor	1.147	0.541	4.498	**0.034**	3.147	1.091	9.080
Times of hospitalization of acute attack of CP, n (%)							
< 3 times	Reference	Reference	Reference	Reference	Reference	Reference	Reference
≥ 3 times	0.052	0.225	0.054	0.817	1.054	0.678	1.638
Pain as first symptoms, n (%)	-0.049	0.341	0.021	0.886	0.952	0.488	1.858
Frequency of abdominal pain during the last 12 months, n (%)							
< 3 times	Reference	Reference	Reference	Reference	Reference	Reference	Reference
≥ 3 times	0.340	0.221	2.364	0.124	1.405	0.911	2.167
Constant pain	1.719	0.565	9.255	**0.002**	5.576	1.843	16.873
VAS-P, n (%)							
0–2 points	Reference	Reference	Reference	Reference	Reference	Reference	Reference
3–6 points	0.494	0.273	3.278	0.070	1.639	0.960	2.797
7–10 points	0.669	0.289	5.361	**0.021**	1.952	1.108	3.437
**Depression**							
Age, y (mean [SD])	-0.001	0.004	0.094	0.760	0.999	0.990	1.007
Educational level, n (%)							
University or above	Reference	Reference	Reference	Reference	Reference	Reference	Reference
Junior high school - Senior High School	0.338	0.174	3.759	0.053	1.403	0.996	1.974
Primary school	0.190	0.298	0.406	0.524	1.210	0.674	2.171
Perceived support from family and friends, n (%)							
High	Reference	Reference	Reference	Reference	Reference	Reference	Reference
Low/Moderate	0.754	0.340	4.926	**0.026**	2.126	1.092	4.139
Previous health status, n (%)							
Good	Reference	Reference	Reference	Reference	Reference	Reference	Reference
Moderate	0.282	0.173	2.642	0.104	1.326	0.944	1.862
Poor	1.447	0.546	7.007	**0.008**	4.248	1.456	12.399
Subjective disease coping level, n (%)							
Good	Reference	Reference	Reference	Reference	Reference	Reference	Reference
Moderate	0.435	0.176	6.111	**0.013**	1.545	1.094	2.180
Poor	1.086	0.572	3.608	0.057	2.963	0.966	9.086
Times of hospitalization of acute attack of CP, n (%)							
< 3 times	Reference	Reference	Reference	Reference	Reference	Reference	Reference
≥ 3 times	0.184	0.185	0.986	0.321	1.202	0.836	1.729
Pain as first symptoms, n (%)	0.029	0.270	0.012	0.914	1.030	0.606	1.748
Frequency of abdominal pain during the last 12 months, n (%)							
< 3 times	Reference	Reference	Reference	Reference	Reference	Reference	Reference
≥ 3 times	0.341	0.188	3.284	0.070	1.406	0.973	2.033
Constant pain	1.307	0.606	4.653	**0.031**	3.694	1.127	12.108
VAS-P, n (%)							
0–2 points	Reference	Reference	Reference	Reference	Reference	Reference	Reference
3–6 points	0.504	0.223	5.122	**0.024**	1.655	1.070	2.560
7–10 points	0.538	0.240	5.039	**0.025**	1.712	1.071	2.739

Abbreviations: CI, confidence interval; CP, chronic pancreatitis; VAS-P, visual analogue scale of pain; OR, odds ratio; SE, standard error

In terms of depression, age (P = 0.012), educational level (P = 0.025), perceived support from family and friends (P = 0.002), previous health status (P < 0.001), subjective disease coping level (P < 0.001), times of hospitalization of acute attack of CP (P = 0.001), first symptom (P = 0.002), frequency of abdominal pain (P < 0.001), and pain severity (P < 0.001) showed significant differences between depressed and non-depressed patients **(**Table 2**)**. Multivariable logistic regression analysis indicated that moderate or low level of social support from family and friends (P = 0.026) and moderate subjective disease coping level (P = 0.013) were related factors for depression in CP patients, and compared to those with good previous health status, patients with poor previous health status (P = 0.008) were more likely to report depression. In addition, constant pain (P = 0.031), moderate (VAS-P: 3–6 points) (P = 0.024) or severe pain (VAS-P: 7–10 points) (P = 0.025) were thought to be independent risk factor for depression symptom in CP patients **(**Table 3**)**.

### Relationship between anxiety, depression and coping style

A correlation analysis was performed to explored the relationship between anxiety, depression, and coping style (Table 4**)**. The result showed that SAS scores were significantly negatively correlated with mature coping styles: problem solving (-0.353) and seeking for help (-0.254), and significantly positively correlated with immature coping styles: self-blame (0.525), fantasy (0.382), repression (0.392) and rationalization (0.313). Similar to anxiety, significant negative correlations were found between SDS scores and problem solving (-0.383), seeking for help (-0.271), and there were significant positive correlations between SDS scores and self-blame (0.484), fantasy (0.296), repression (0.355) and rationalization (0.293) (all P < 0.001).

**Table 4:** The correlation analysis of anxiety, depression, and coping style

	SAS score		SDS score	
Variables	*r*	*P*	*r*	*P*
Coping style				
Problem solving	-0.353	**< 0.001**	-0.383	**< 0.001**
Self-blame	0.525	**< 0.001**	0.484	**< 0.001**
Seeking for help	-0.254	**< 0.001**	-0.271	**< 0.001**
Fantasy	0.382	**< 0.001**	0.296	**< 0.001**
Repression	0.392	**< 0.001**	0.355	**< 0.001**
Rationalization	0.313	**< 0.001**	0.293	**< 0.001**

Abbreviations: SAS, Self-rating Depression Scale; SDS, Self-rating Depression Scale

## Discussion

CP is a clinically rare disease of the digestive system. Currently, most of the studies have focused on the pathogenesis, clinical symptoms and treatment of CP, but less on the mental health of CP patients and its influencing factors. To the best of our knowledge, this study is the first to investigate the incidence of anxiety and depression in East Chinese CP patients and to analyze their relationship with patient demographic characteristics, clinical characteristics of CP, and coping styles. Our findings suggested that a relatively high proportion of Chinese CP patients have obvious symptoms of anxiety and depression, and that anxiety and depression are significantly associated with patients’ previous health status, frequency of abdominal pain episodes, pain severity, and level of disease coping.

In our study, more than one-fifth (22.64%) of CP patients reported symptoms of anxiety and nearly 40% (38.61%) of patients had depressive symptoms. However, this result is significantly lower than the results of a previous five-year population-based cohort study, in which the prevalence of anxiety and depression among CP patients in the United States was 36.8% and 46.8%, respectively[23]. The differences in results may arise from the following aspects. First, although the study was based on a longer and larger study cohort, it did not explicitly disclose the assessment instruments for anxiety and depression due to its retrospective nature. Differences in morbidity may stem from the differences in assessment performance between different measurement tools. In addition, since the data in their study was sourced from public database, the consistency of records between different institutions could not be guaranteed [24]. Second, in terms of the epidemiology of CP, unlike Western countries, alcoholism only accounts for about 20% of the causes of CP in Chinese patients [25]. Since alcohol abuse is considered to be one of the important risk factors for increased pain and increased stone formation in CP patients [26, 27], the differences in the etiology of CP between East and West and the different severity of the disease caused by it may contribute to the differences in the incidence of anxiety and depression. In addition, Shah et al. found that the prevalence of opioid dependence among CP patients in the United States was 48.87%, and opioid dependence was associated with a higher incidence of anxiety and depression [28]. However, the use of opioids in China is quite conservative due to medical regulations [29]. Since patients cannot easily buy opioid analgesics, most of them tend to go to the hospital soon after the onset of symptoms. In this study, patients with constant pain only accounted for 2.50% of the total participants, significantly lower than the 45.25% incidence of constant pain in a previous study [30]. Therefore, differences in pain types, analgesic medication use, and health care habits between Eastern and Western CP patients may explain the lower incidence of anxiety and depression in this study. In addition, the incidence of depression among CP patients in our study is also lower than that in a study from India, in which the incidence of depression in Indian CP patients is 46.77% [31]. In their study, researchers used the Beck depression inventory (BDI) to measure the depression level of patients, while in our study, we used the SDS. Through literature search, we found that existing research evidence have showed that compared with other self-reported depression assessment tools, when measured with BDI scale, the incidence of depression was significantly higher [32, 33]. Therefore, we believe that the use of different measuring tools of depression may be the reason for the different incidence of depression in the two studies. Our study provided preliminary evidence for the epidemiology of anxiety and depression in Chinese CP patients. It is expected that future studies can recruit more patients, incorporate more demographic and clinical characteristics, and use a variety of other anxiety and depression measurement tools to further explore the prevalence and risk factors of anxiety and depression in Chinese CP patients on the basis of this study.

Patients with CP often experience recurrent abdominal pain, weight loss, steatorrhea, and other comorbidities such as diabetes, pancreatic duct stones, pancreatic duct stenosis, and a higher risk of pancreatic cancer. These physical and psychological stressors and stressful events may jointly lead to the development of anxiety and depression [34]. Pain and psychological comorbidities play an important role in the progression of the disease course among CP patients. In this study, we found that higher VAS-P scores (7-10points) and more frequent abdominal pain episodes in the past 12 months (constant pain) were significantly associated with the occurrence of anxiety and depression in CP patients. Existing studies have reported that the existence of psychological comorbidities in CP patients may aggravate their pain symptoms and affect their pain experience [35, 36], and pain was considered to be closely related to adverse outcomes such as weakness, decreased quality of life, and increased disability [14, 37]. Furthermore, Kroenke et al. found that pain and depression had a bidirectional effect, with changes in the severity of one symptom likely to have the same effect on the other [38]. Therefore, the interventions for pain and prevention for psychological disorders are particularly important. However, so far, there is still a lack of observational and interventional studies on psychological disorders such as anxiety and depression in CP populations. We recommended that, for patients with mild pain, screening for psychological disorders can be carried out early to prevent anxiety or depression symptoms from aggravating the pain experience of patients, and for CP patients with severe pain, it is very meaningful to conduct psychological counseling for patients while carrying out pain intervention, which can relieve their anxiety and depression symptoms, and then assist in pain relief.

Coping style is considered a habitual tendency of individuals to solve problems and a common strategy for coping with stressful events [39]. Studies have shown that coping styles were significantly related to anxiety and depression [40]. This result was also validated in our study. In our study, low levels of subjective disease coping were found to be independent risk factors for anxiety and depression in CP patients. Subsequent further correlation analysis of coping styles with anxiety and depression scores showed that mature coping styles (Problem solving, Seeking for help) had a positive impact on anxiety and depression scores, while immature coping styles (Self-blame, Fantasy, Repression, Rationalization) had negative effects on anxiety and depression scores. Therefore, for medical service providers, it is necessary and important to strengthen health education for CP patients, guide them to adopt mature coping methods, establish the concept of “Solving Rather than Escaping” when facing the difficulties and pressures brought by the disease, and instruct them to actively try to seek others’ help.

This study had some limitations. First, the number of relevant factors included in this study was limited and measured in a crude manner. Some variables in our study, such as health status and social support, were not measured using specific assessment scales. Based on the results of this study, future researches can select these professional scales to further explore the specific relationships between the above variables and anxiety and depression in CP patients. Second, we only recruited CP patients admitted to our hospital for treatment. On the one hand, since our center is the largest CP diagnosis and treatment center in China, the patients in this study were more seriously ill compared to patients from other medical institutions and patients who were not hospitalized, and thus may lead to an overestimation of the incidence of anxiety and depression. On the other hand, the patients in our center mainly come from Shanghai, Jiangsu, Zhejiang, Anhui and Jiangxi proviences in eastern China[41]. Due to the better economic level and educational resources in eastern China compared with the central and westernChina, as well as the important impact of patients’ educational level and disease knowledge on the patients’ psychological comorbidities, the epidemiological evidence of anxiety and depression in patients with CP in our study may still be biased from the overall status of anxiety and depression in patients with CP in China. It is suggested that future studies should not be limited to a single-center clinical setting, but could conduct multicenter and even community-based epidemiological investigations on the prevalence of anxiety and depression in Chinese CP patients. Although this study has the above limitations, it is undeniable that it is still the first and largest study on the epidemiological status of anxiety and depression in patients with CP in China, filling the gap in the study of psychological comorbidities in Chinese CP patients.

## Conclusion

Anxiety and depression are common in East Chinese CP patients. Our study found that CP patients with anxiety and depression had worse previous health status and subjective disease coping levels, higher frequency of abdominal pain episodes, and greater pain severity. Anxious patients were more likely to be female, and depressed patients had lower levels of social support. In addition, mature coping styles were found to have a positive impact on anxiety and depression. Our findings provide preliminary evidence for the investigation of the epidemiology and influencing factors of anxiety and depression in Chinese CP patients, and provide a reference for the management of anxiety and depression. It is hoped that the screening and early intervention of psychological comorbidities in CP patients can be strengthened in future clinical practice to reduce the level of anxiety and depression in Chinese CP patients.

## Data Availability

The datasets used and/or analyzed during the current study are available from the corresponding author on reasonable request.

## Abbreviations

CP: chronic pancreatitis
CSQ: Coping Style Questionnaire
SAS: Self-rating Depression Scale
SD: standard deviations
SDS: Self-rating Depression Scale
VAS-P: visual analogue scale of pain
